# Advancing the National Immunization Program in an era of achieving universal vaccine coverage in China and beyond

**DOI:** 10.1186/s40249-024-01192-6

**Published:** 2024-03-13

**Authors:** Shu Chen, Lance E. Rodewald, Anna Heng Du, Shenglan Tang

**Affiliations:** 1 ARC Centre of Excellence in Population Ageing Research (CEPAR), University of New South Wales, Sydney, Australia; 2 School of Risk and Actuarial Studies, University of New South Wales, Sydney, Australia; 3 Department of the National Immunization Program, Chinese Center for Disease Control and Prevention, National Immunization Program, China CDC, Beijing, China; 4 China Country Office, Bill & Melinda Gates Foundation, Beijing, China; 5 Duke Global Health Institute, Duke University, Durham, NC USA; 6 Global Health Research Center, Duke Kunshan University, No. 8 Duke Avenue, Kunshan, 215316 Jiangsu China; 7 SingHealth Duke-NUS Global Health Institute, Duke-NUS, Singapore, Singapore

**Keywords:** National Immunization Program, New vaccine introduction, Universal vaccine coverage, Health system strengthening

## Abstract

**Background:**

Immunization is a cornerstone of public health. Despite great success, China’s National Immunization Program (NIP) faces challenges, such as the integration of several World Health Organization-recommended vaccines and other systemic issues. The Innovation Laboratory for Vaccine Delivery Research (VaxLab), supported by the Bill & Melinda Gates Foundation and established in 2021 at Duke Kunshan University, focuses on enhancing China’s NIP through research and policy advocacy. This editorial aims to summarize the key findings of the manuscripts published in the collection contributed by VaxLab team and set the future research agenda.

**Key findings:**

The collection contains eleven manuscripts discussing China’s immunization landscape and strategies to improve coverage, particularly for non-NIP vaccines like human papillomavirus vaccine (HPV), pneumococcal conjugate vaccine (PCV), *Haemophilus influenzae* type b vaccine (Hib), and rotavirus vaccines. Key findings include: (i) The COVID-19 vaccination campaign demonstrated China’s capacity for rapid, large-scale immunization efforts, suggesting potential for broader vaccine coverage improvements; (ii) Efforts in combating cervical cancer through the HPV vaccine indicate progress but also highlight challenges like vaccine supply and equitable access; (iii) The lag in adopting higher-valent paediatric combination vaccines in China needs attention to address regulatory and health system hurdles; (iv) Disparities in access to non-NIP vaccines underscore the need for government initiatives to improve vaccine coverage, especially for remote areas and marginalized populations; (v) Original studies emphasize the influence of caregivers’ knowledge, health workers’ financial incentives, and concerns about vaccine efficacy on immunization rates; (vi) Case studies from the Weifang City of China and Indonesia to introduce PCV offer insights on successful vaccine introduction strategies and the impact of innovative financing and government support.

**Conclusion:**

The articles emphasize the need for government leadership, strategic policymaking, and public awareness to enhance vaccine coverage and equity. The VaxLab will continue strengthening China’s NIP by focusing on vaccine financing, emphasizing diversity, equity, and inclusion, and improving maternal vaccination coverage. Research will extend to Southeast Asian and Western Pacific regions, especially in middle-income countries facing challenges in vaccine financing and delivery. The collective efforts outlined in this collection show a commitment to evolving and adapting immunization strategies to meet global health goals and to provide equitable access to vaccines for all.

**Graphical Abstract:**

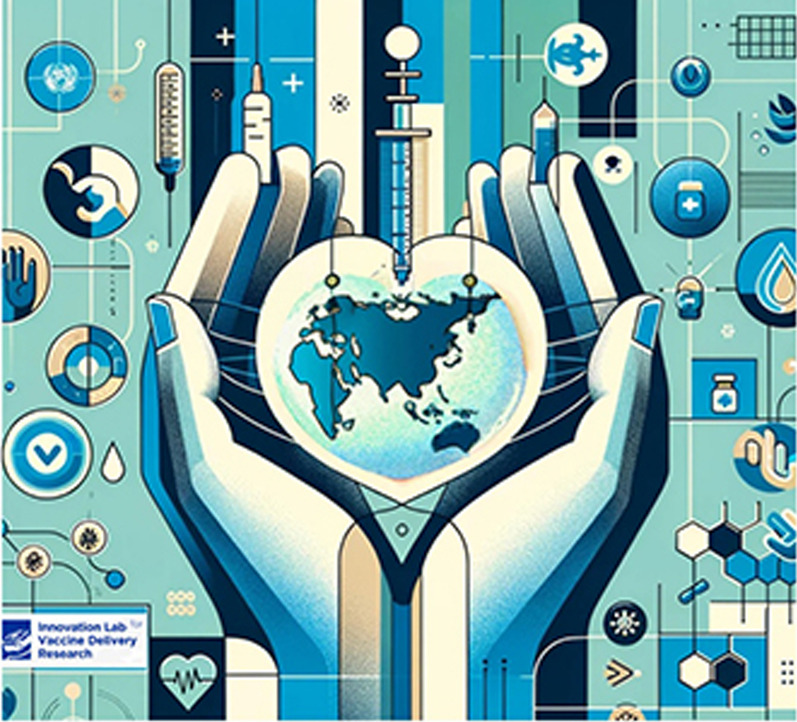

## Background

Public health’s foundation has been significantly bolstered and strengthened by immunization programs implemented by national governments, protecting people, especially children, from a wide array of vaccine-preventable diseases (VPDs). This year marks the 50th anniversary since the launching of the Expanded Programme on Immunization (EPI) of the World Health Organization (WHO) in 1974 [1]. China’s National Immunization Program (NIP) was initiated in 1978 and has been a model of public health success, having achieved over 95% coverage for children under six years old with 14 vaccines targeting 15 diseases [2]. It reflects a monumental public health achievement in the fight against infectious diseases. However, despite these successes, China’s NIP faces ongoing challenges, particularly expanding the list of free vaccine offerings [3]. Since 2008, NIP has not introduced any of the newer vaccines that are recommended by the WHO for inclusion in all countries’ national immunization programs. This stagnation has resulted in limited access to critically important vaccines like pneumococcal conjugate vaccine (PCV), human papillomavirus vaccine (HPV), *Haemophilus influenzae* type b vaccine (Hib), and rotavirus vaccines [4]. While the better-off population in China can afford to pay out of pocket for these non-program vaccines, people from low-income groups often cannot afford them. The disparity in access to these vaccines is clearly evident in terms of coverage among different socio-economic groups, highlighting the need for a more inclusive approach to financing and organizing immunization service delivery [3, 4].

Immunization Agenda 2030 (IA2030) is an ambitious global strategy adopted by the WHO and its partners that aims to reduce the number of vaccine-preventable deaths and diseases significantly and achieve universal coverage with WHO-recommended vaccines. IA2030's core components and strategic priorities emphasize the need for strengthening health systems and integrating an inclusive and life-course approach to immunization [5]. Improving vaccine coverage will also contribute to achieving the health-related United Nations Sustainable Development Goals (SDGs), particularly goals for reducing child mortality (Target 3.2), combating communicable diseases (Target 3.3), and strengthening the capacity of all countries for early warning, risk reduction, and management of national and global health risks (Target 3.d) [6].

As one of the most populous countries in the world, with a large number of children to be vaccinated each year, improving vaccine coverage in China will significantly contribute to achieving the IA2030 vision and the health-related SDGs. A systematic and comprehensive approach is needed to strengthen China’s NIP. Relevant policies and solutions should target health governance, financing, workforce, information systems, and vaccine delivery to improve vaccination coverage levels [3].

With support from the Bill & Melinda Gates Foundation, the Innovation Laboratory for Vaccine Delivery Research (VaxLab) was established in 2021 at the Global Health Research Center of Duke Kunshan University. The aim of VaxLab is to strengthen China’s NIP via generating high-quality evidence and conducting effective policy advocacy activities. The eleven manuscripts published in this thematic series of the journal *Infectious Diseases of Poverty*, “Advancing the National Immunization Program in China: Making it More Effective and Sustainable,” are outputs emanating from ongoing work of VaxLab. They provide an in-depth look at the strides made and the hurdles faced in the realm of vaccine delivery and coverage in China. In this editorial, we summarize these journal contributions and offer an overview of the state of immunization in China, while setting the stage for future research and policy advocacy to achieve universal vaccine coverage in China.

## Key findings

The collection features eleven articles. Ten provide different perspectives on China's immunization landscape, with particular focus on improving coverage levels of four key non-program vaccines: HPV, PCV, Hib, and rotavirus. One of the articles shares experiences and lessons learnt from Indonesia’s introduction of PCV. The collection has a diversity of manuscripts, including three commentaries, four original studies, one scoping review, and three case studies.

### Commentaries

The three commentaries address pivotal aspects of vaccination efforts in China, encompassing the COVID-19 vaccination campaign, HPV vaccination initiatives, and the adoption of higher-valent paediatric combination vaccines. Dr. Lance Rodewald highlights China’s swift mobilization of resources to achieve widespread COVID-19 vaccine coverage, advocating for the application of this infrastructure and experience to sustain high immunization rates as per IA2030 objectives, across all age groups for all WHO-recommended vaccines [7]. Prof. Fanghui Zhao and colleagues discuss the advances and challenges in China’s approach to HPV vaccination as a measure against cervical cancer, acknowledging significant progress but also calling attention to the need for stable vaccine supply, fair pricing, and a focus on marginalized populations to ensure equitable vaccine access [8]. A collaborative analysis by Prof. Shenglan Tang’s Duke Kunshan University team and Prof. Fuqiang Cui’s Peking University team examines the sluggish uptake of higher-valent paediatric combination vaccines in China, citing legislative and public awareness barriers while recommending a comprehensive promotion strategy that includes stricter vaccination law enforcement, schedule alignment, and enhanced vaccine research and development [9]. Together, these commentaries underscore the necessity of a concerted effort to enhance vaccine financing, services, access, and regulatory environment to stimulate vaccine innovation, so as to improve vaccine coverage in China.

### Scoping review

The collection includes one scoping review led by Prof. Xiaohua Ying from Fudan University on coverage of the four underutilized non-NIP vaccines in China mentioned above—HPV, PCV, Hib, and Rotavirus—that are not part of China’s NIP but are primarily financed through out-of-pocket payment by families [4]. The authors identify gaps in coverage and the need to tackle issues related to access, affordability, acceptance, awareness, and activation. They advocate for a strategic government initiative to methodically incorporate these vaccines into China’s NIP and emphasize the importance of addressing disparities in vaccine access, particularly focusing on marginalized populations and less developed areas [4].

### Original studies

The collection includes four original studies focusing on different immunization topics among diverse populations and regions, with a common aim to improve non-NIP vaccines coverage in China. These topics include vaccine coverage among the migrant and left-behind families, pentavalent vaccination coverage, *Streptococcus pneumoniae* nasopharyngeal carriage among children under five, and public health workers’ recommendations for non-NIP vaccines.

Original evidence on the low coverage of non-NIP vaccines has been generated. The lower vaccination coverage of non-NIP vaccines among migrant and left-behind families reported in the study led by Dr. Xiaolin Xu shows that factors such as the ages of children and caregivers, family roles, and caregivers’ knowledge about vaccines significantly influence vaccination rates [10]. It calls for specific interventions to enhance immunization information dissemination and satisfaction among these vulnerable groups, aiming to bridge the coverage gap with local urban families. The study led by Prof. Guohong Li focuses on pentavalent vaccine, analyzing its low coverage in Southern China. They identified key factors influencing caregiver decisions to vaccinate, including concerns about vaccine efficacy, manufacturer reputation, and the complexity of immunization schedules [12]. They show that urban–rural disparities exist, with urban caregivers displaying more concern about cost and convenience.

Consistent with the low coverage of PCV vaccine, evidence shows the carriage rate of *Streptococcus pneumoniae* is not low. The study led by Dr. Jiang Wu obtained nasopharyngeal swab samples from 2333 children in Hainan and found *Streptococcus pneumoniae* in 30.4% of the samples, spanning 29 serotypes. PCV13 vaccine covered 60.9% of the serotypes found in their study, and PCV13-vaccinated children had lower carriage rates [13]. Risk factors for carriage included attending day-care and having siblings, whereas children of more educated mothers, those living in urban areas and fully vaccinated with PCV13 had lower carriage rates, suggesting that the vaccine’s introduction could markedly reduce pneumococcal infections.

As medical and public health doctors’ recommendations may play a pivotal role in increasing the uptake of non-NIP vaccines, the study led by Prof. Hai Fang further provides insight into the behaviours of public health workers in recommending non-NIP vaccines, revealing a direct correlation between their recommending practices and financial incentives [11]. In addition, perceptions of vaccine safety play a significant role. Their study underscores the need for policies that encourage health workers to advocate for vaccinations, potentially through financial motivation, to achieve broader public health goals.

Together, these studies provide empirical evidence on the low coverage of non-NIP vaccines, especially among vulnerable population, and a comprehensive analysis of current challenges and factors affecting immunization service delivery in China. They highlight the need for nuanced strategies to address gaps in vaccine coverage and acceptance, and the potential impact of educational and policy interventions in improving public health outcomes.

### Case studies

The collection presents three case studies detailing the experiences and insights gained from pilot programs aimed at introducing HPV and PCV vaccines in China and Indonesia, providing valuable experience and lessons for China and other middle-income countries (MICs).

China started to introduce free HPV vaccine in 2021 in selected pilot cities as part of the Healthy City Innovation Pilot Program. In contrast, few cities have tried to introduce PCV vaccine into the local immunization program. The two case studies on HPV vaccine introduction in Shenzhen City and PCV introduction in Weifang City are snapshots of the ongoing new vaccine introduction effort in China. A case study on the HPV vaccine pilot in Shenzhen City of China, led by Dr. Yueyun Wang, credits its success to strong government support, strategic funding, multi-sectoral effort, and effective communication, while also recognizing challenges such as vaccine hesitancy and service quality, suggesting that ongoing innovation and partnerships are essential for wider vaccine uptake [14]. The PCV case study, led by Prof. Luzhao Feng, showcases an innovative PCV13 vaccination initiative in Weifang City, Shandong Province, China, where offering the first dose for free to young children and using commercial insurance for older children led to increased vaccination rates. Despite improvements, the authors acknowledge the need to explore further innovative financing and possibly reduced-dose schedules to reach coverage levels comparable to developed countries [15].

Indonesia started to introduce PCV vaccine in 2017, with the support from the Clinton Health Access Initiatives (CHAI) team. This case study provides a comprehensive overview of the strategic approaches that overcame hurdles in the introduction of PCV vaccine in Indonesia from 2017 to 2022. Key to the program’s success were high-level political support, evidence-based decision-making, and a sustainable vaccine procurement and distribution system [16]. This case is particularly instructive for nations transitioning from The Vaccine Alliance (Gavi) support and grappling with similar challenges in vaccine finance and delivery.

Collectively, these studies underscore the critical role of governmental support, financial strategies, stakeholder collaboration, and communication in the successful introduction and scaling up of vaccination programs in MICs, while also highlighting the importance of addressing vaccine hesitancy and improving service quality to achieve broad and sustained coverage.

### Lessons learned and the way forward

These eleven articles provide insight into successes and challenges of vaccine implementation across different regions and demographics. Several of the articles highlight the importance of government leadership, strategic policy making, financial incentives, and public awareness in improving vaccine coverage and equity in China. Considering the rapidly changing landscape of vaccine technology and public health policies globally and in China, supporting research on implementation strategies and practices from a system perspective is imperative. Hence, VaxLab will continue working to strengthen China’s NIP and accelerate the inclusion of WHO-recommended vaccines into the NIP. Specifically, VaxLab’s future research endeavours will focus on three key areas:

- Improving vaccine financing. VaxLab will work to analyse and compare government investment in public health programs and will conduct modelling analyses to estimate saved costs if health insurance could pay for the four non-NIP vaccines—PCV, HPV, Hib, and rotavirus vaccines—and will analyze costs and prices of existing and potentially-included NIP vaccines for market shaping and business sustainability of NIP vaccine suppliers. VaxLab will also support a randomised control trial to evaluate the effect of a pay-it-forward strategy, which is an innovative vaccine financing technique designed to increase HPV vaccine coverage among school girls aged 15–18 years.
- Emphasizing diversity, equity, and inclusion (DEI) in immunization. A focus on DEI is crucial for developing inclusive strategies that ensure equitable access to vaccines for all segments of the population. VaxLab will support several studies of access to vaccines in remote areas and low-income areas (such as western China) and among vulnerable populations (such as left-behind children), to explore effective ways to improve vaccine coverage and regional equity.
- Maternal vaccination. Enhancing vaccine uptake among pregnant women is a vital component of maternal and neonatal health. However, awareness and acceptance of maternal vaccination are very low in China, resulting in very limited uptake of vaccines recommended for pregnant women. VaxLab will support a study led by Fudan University to map available evidence about maternal vaccination, including coverage and research gaps, that will explore effective ways to increase awareness and acceptance of maternal vaccination in China.

VaxLab will expand its geographic interest to include countries in the Southeast Asian and Western Pacific WHO regions, especially in those Gavi-ineligible MICs that face the greatest challenges in financing and delivering vaccines [17]. Research collaborations and capacity building activities will focus on strengthening the NIPs via a health system approach, and facilitating quality and accelerated new vaccine introduction. Good practices, challenges, and lessons learnt will be shared through joint efforts to improve vaccine coverage in China and other MICs.

## Conclusions

This thematic series on China’s NIP provides an overview of many successes and challenges faced in immunization in China. It highlights the need for continuous research and policy adaptation to ensure equitable and effective immunization strategies, particularly in areas like diversity, equity, and inclusion and maternal vaccination. Future VaxLab research will contribute significantly to this endeavour, aiming to enhance health outcomes through improved immunization practices. These efforts will not only benefit China but will also provide valuable lessons and models for other developing countries striving to improve their immunization programs.

## Data Availability

Data sharing is not applicable to this article as no datasets were generated or analyzed during the current study.

## Abbreviations

CDC: Center for disease control and prevention
CHAI: Clinton health access initiatives
DEI: Diversity, equity and inclusion
EPI: Expanded program on immunization
Gavi: The vaccine alliance
Hib: *Haemophilus influenzae* type b
HPV: Human papillomavirus
IA2030: Immunization Agenda 2030
IPV: Inactivated poliovirus vaccine
MICs: Middle-income countries
NIP: National immunization program
PCV: Pneumococcal conjugate vaccine
SDGs: Sustainable development goals
VaxLab: Innovation laboratory for vaccine delivery research
VPD: Vaccine-preventable disease
WHO: World Health Organization
